# Anæsthesia vs Asphyxia

**Published:** 1895-09

**Authors:** S. A. Aykroyd

**Affiliations:** Kingston, Ont.


ARTICLE III.

### ANÆSTHESIA VS. ASPHYXIA.

BY S. A. AYKROYD, D. D. S., L. D. S., KINGSTON, ONT.

Read before the Eastern Ontario Dental Association.

Having seen the question, "What is the difference between Anæsthesia and Asphyxia?" in the *Dominion Dentel Journal*, I became interested and took to discussing it with my co-laborer, the editor of the "Question Drawer," who suggested that I prepare a paper for this meeting on the subject.

If you are sorely afflicted by what I say, blame not me but the instigator of the deed. Jokes aside, gentlemen, I have been reading some and have gleaned a little from off the field of knowledge *ie* the question, and with the hope that I may be able to present you with some food for thought, or, at least, afford a basis for discussion. I undertake this task. He who deals with life should know the laws that govern it. But what are laws and what is life? may be asked.

Laws are relations between cause and effect. The same cause will always produce the same effect, if that on which yon operate is the same. If this were not so, science would be impossible. Laws are eternal and fixed principles, to be in harmony with which is life, the opposite is death. It remains for man, then, to discover the laws of life and obey them.

Life is a phenomenon which as yet has not been satisfactorily defined. I asked the professor of biology in Queen's University, What is life? After several days he replied, "I can't answer your question." Life has been expressed by Savory as "A state of dynamical equilibrium." Life, however, does not stand alone; it is but a special manifestation of transformed force. Anæsthesia has been variously defined. A definite understanding of what is meant by it is a matter of no small importance to those whose mission it is to relieve pain and save life.

Webster says, "An anæsthetic is that which produces insensibility." Our own Dr. Teskey says, "Anæsthesia is deprivation of the sense of feeling from any cause." Surely such definitions cannot be scientific, for if they are then a club, a rope or a cannon ball would be an anæsthetic, and death itself, anæsthesia. Dr. Thomas, of Philadelphia, says, "Anæsthesia is an unconscious condition produced by the inhalation of various drugs, the forces of life being maintained in the meantime." This is a more comprehensive definition, but still we are not satisfied. Dr. Hayes, in the *Dental and Surgical Microcosm*, from which I take the liberty to quote freely, says, "Æsthesia is a condition of sensibility, but in order to realize sensation the force and functions of life must be continued."

Anæsthesia is the opposite of æsthesia, and is a condition of insensibility, and consequently in this condition there must also be in continuance the force and functions of life. Therefore the correct definition, according to Dr. Hayes, is, "Anæsthesia is a physical condition in which the force and functions of life are in continuance under modifications whereby absensation is produced." This, it seems to me, comes nearer to a scientific definition than any other I have seen. The force of life is free oxygen, or rather, according to recent discoveries in science, electricity is the great force of life, and that oxygen is the carrier of this electricity.

Dr. W. B. Richardson, an English physiologist, has conducted experiments showing that pure oxygen becomes devitalized by repeated inhalations that in breathing the oxygen had undergone some change unknown to the chemist, and that if the oxygen be electrically charged it is revitalized and will again support life.

The chief functions of life we are interested in during anæsthesia are circulation and respiration. To properly understand the science of anæsthesia, we must recognize the double circulation in every animal, the one dependent upon the other. Arterial and venous circulation is as dependent upon neural circulation in the nervous system as life is dependent upon the functions of life. The brain is the heart of the nervous system, and it might be designated the animal electric storage battery.

From the basic element *free oxygen animo electricity* is generated by the pneumogastric glands of the lungs and the cerebral glands of the lungs and the cerebral glands of the brain, and stored in this storage battery to be sent out through the motor nerves as a force to move the organs of the body. This circulation through the nervous system brings back to the brain the greater portion of the electricity, through the sensory nerves, and thus information and impressions are received from the material world by the mind or spirit of man.

Interrupt or arrest this circulation in the nervous system, and you suspend sensation and the force and functions of life, and then you have the first stage of death. From what has been said it is obvious that a true anæsthetic must contain sufficient free-oxygen to fill this storage battery with animo-electricity, or nerve-vital fluid, that the functions of life may not be interfered with. In administering an anæsthetic, death can only be caused by either asphyxia or by shock. Shock is produced by the mind or by the intended anæsthetic agent, or by both acting simultaneously.

What is asphyxia, and how is it produced? Blood which contains a normal proportion of oxygen excites the respiratory centre, and consequently the respiratory muscular movements are normal. A deficiency of oxygen gives a condition of muscular movements called dyspnœa (difficult breathing). When respiration is stopped by interference with the passage of air to the lungs by supplying air devoid of oxygen, a condition ensues which passes rapidly from the state of dyspnœa to what is termed asphyxia, or suffocation, which quickly ends in death.

The ways by which asphyxia may be produced are numerous; for example, by prevention of the due entry of oxygen into the blood either by obstruction of the respiratory passages or by the introduction of gas devoid of oxygen, or of a gas containing oxygen which is not *free*, and, consequently, a due interchange in the blood cannot take place (a gain of oxygen and a loss of carbonic acid).

The symptoms of asphyxia are: violent action of the respiratory muscles and more or less of all the muscles of the body, lividity of the skin and all other muscular parts; the veins become distended and the tissues seemed gorged with blood, convulsions and insensibility, which is quickly followed by death. The conditions which accompany these symptoms are: (1) More or less interference with the passage of the blood through the pulmonary blood-vessels; (2) Accumulation of the blood in the right side of the heart and in the systemic veins; (3) Circulation of impure blood in all parts of the body.

The cause of these conditions and the manner in which they act so as to be incompatible with life may be briefly considered: (1) By the violent and convulsive action of the expiratory muscles, pressure is directly made on the lungs, and the circulation through them is proportionately interfered with. This is the direct cause of the accumulation of blood in the right side of the heart. (2) The vaso-motor centres, stimulated by blood deficient in oxygen, cause contraction of all the small arteries, with increase of arterial tension, and as an immediate consequence the filling of the systemic veins. The increased arterial tension is followed by inhibition of the action of the heart, which, contracting less frequently and gradually enfeebled also by deficient supply of oxygen, becomes over distended by blood which it cannot expel. At this stage the left as well as the right cavities are distended with blood. The ill effects of these conditions are paralysis of the muscles of the heart by over-stretching, venous congestion and consequent interference with the function of the higher nerve-centres, especially the medulla oblongata. (3) The passage of non-aerated blood through the lungs and its distribution over the body are events incompatible with life for more than a few minutes. The rapidity with which death ensues in asphyxia is due to the effect of non-oxygenated blood on the medulla oblongata, and through the coronary arteries on the muscular substance of the heart.

Experiments have been performed on the lower animals, and it has been found in the case of the dog, during simple asphyxia, that is by simple privation of air by plugging the trachea, that the average duration of the respiratory movements was four minutes five seconds. The average duration of the heart's action was seven minutes eleven seconds, recovery not taking place after the heart ceased. Thus we see that asphyxia is a physical condition radically differing from that of anæsthesia.

In anæsthesia the force and functions of life are in continuance under modification, whereby absensation is produced by medico-chemical agents, narcotizing the muscular tissues and the nerve filaments, thereby interrupting the neural current in the sensory nerves. In asphyxia the force and functions of life are suspended or abrogated whereby absensation is produced by the deoxidation of the blood. thus producing insensibility by preventing the generation of nervo-vital fluid and its circulation in the sensory nerves. The anæsthetic agent must have sufficient *free oxygen* to properly support combustion and sustain life. An asphyxiant must be deprived of free oxygen or must not have sufficient to properly support combustion and sustain life. Oxygen chemically combined with any other material element will neither support combustion nor sustain lfe.

Therefore, nitrous oxide gas ($N_2 O$) will no more sustain life than carbonic acid gas, or nitrogen gas, or olyphiant gas, or any other inert gas. While it is maintained that $N_2 O$ will support combustion by applying a lighted taper or a piece of heated charcoal, the fact is lost sight of that there is not heat enough in the human body to decompose the two chemical elements and set the oxygen free, and that it is the free oxygen and not the nitrous oxide gas that supports combustion.

Robert Marston, Lancaster, England, writing in the *Items of Interest*, in substance says: "To suppose that nitrous oxide is an anæsthetic is to assume that it either plays the part of a toxic compound radicle, in the vito-chemical equations of the body, or else that its disassociated elements separately participate in the play of affinities, but, if nitrous oxide disported itself as a toxic compound radicle, its narcotic power would assert itself, as that of chloroform, ether and other narcotics, though atmospheric air were freely admitted with it to the lungs, a result which practitioners know is impossible of attainment. On the other hand, if nitrous oxide became chemically split up during the corpuscular changes, arterialization of the blood would in that case inevitably ensue with abnormal energy, owing to the greater proportion of oxygen which nitrous oxide $N_2 O$ contains, as compared with the proportions of oxygen and nitrogen found in common air $N_4 xO$. That this arterialization does not take place is sufficiently proved by the characteristic lividity of gas patients and other indications."

"Physiological effects are incompatible with the supposition that nitrous oxide is an anæsthetic; appearances distinguish it as a negative asphyxiant.

"When nitrous oxide comes to be regarded as a negative asphyxiant it will then be understood why it has the reputation of being the safest of all, so-called, anæsthetics. The prevailing opinion that it is a narcotic, whose peculiar action distinguishes it so distinctly from the dangerous compounds of its class, appears irrational when the diffusibility and cumulative tendency of all respiratory narcotics is relatively considered. During that kind of anæsthesia which results from oxygen starvation, all the co-existing conditions are relatively connected; their deflection is uniform, and represents a string of alternated causes and effects, whose tension to rebound with reparative co-operation so soon as the swerving power of the asphyxiant is removed, constitutes that ignored cause which distinguishes the safeness of nitrous oxide from the ungovernable capriciousness of every other tabulated 'anæsthetic.'

"The absence of oxygen causes the accumulation of natural products and corbonic acid, which have a toxic action on the nerves, obtunding their sensibility: When chloroform, ether or other anæsthetics are used, the result is different; toxic asphyxiants have a stronger affinity for the complex molecules of nervous tissues than for the crude and comparatively elementary substance of the circulation. Thus they directly attack the fundamental principles which, subservient to the first cause, create and control all the phenoment of life, sometimes paralyzing the vital endowments, even while the respiratory changes are, to all appearances, ordinarily taking place."

Although deaths are rare from nitrous oxide gas, being only one in a million, let us remember, to obtain anything like complete insensibility with it, we must push the patient dangerously near the death-line, and that no anæsthetic agent should be administered without the admission of plenty of free oxygen or atmospheric air.

The time is coming when we shall be able to understand the physics of the breath of life, or of the oxygen of respiration, which is also the oxygen of combustion; how the inrush of oxygen, by way of capillary gates of the lungs and the corpuscles of the blood, to the tissue-cells of all parts of the system, carries a ceaseless volume of vital energy; how this animating and life-maintaining energy is nothing less than electricity of absolute dynamic strength and sureness—every breath, according to its size a definite quantity of vitalizing, heating and sustaining energy, and how the flow and charge of this energy, in all parts of the system, will maintain functions and operate organs which it has, in fact, created.—*Dominion Dental Journal.*

